# Supplementary material for: Topical Wound Treatment with a Nitric Oxide-Releasing PDE5 Inhibitor Formulation Enhances Blood Perfusion and Promotes Healing in Mice
Source: Pharmaceutics. 2022 Oct 31;14(11):2358. doi: 10.3390/pharmaceutics14112358 (PMC9696390; doi:10.3390/pharmaceutics14112358)
Supplement: Supplementary file 1 [file pharmaceutics-14-02358-s001.zip › pharmaceutics-1957616-supplementary.pdf]

Supplementary Figures:

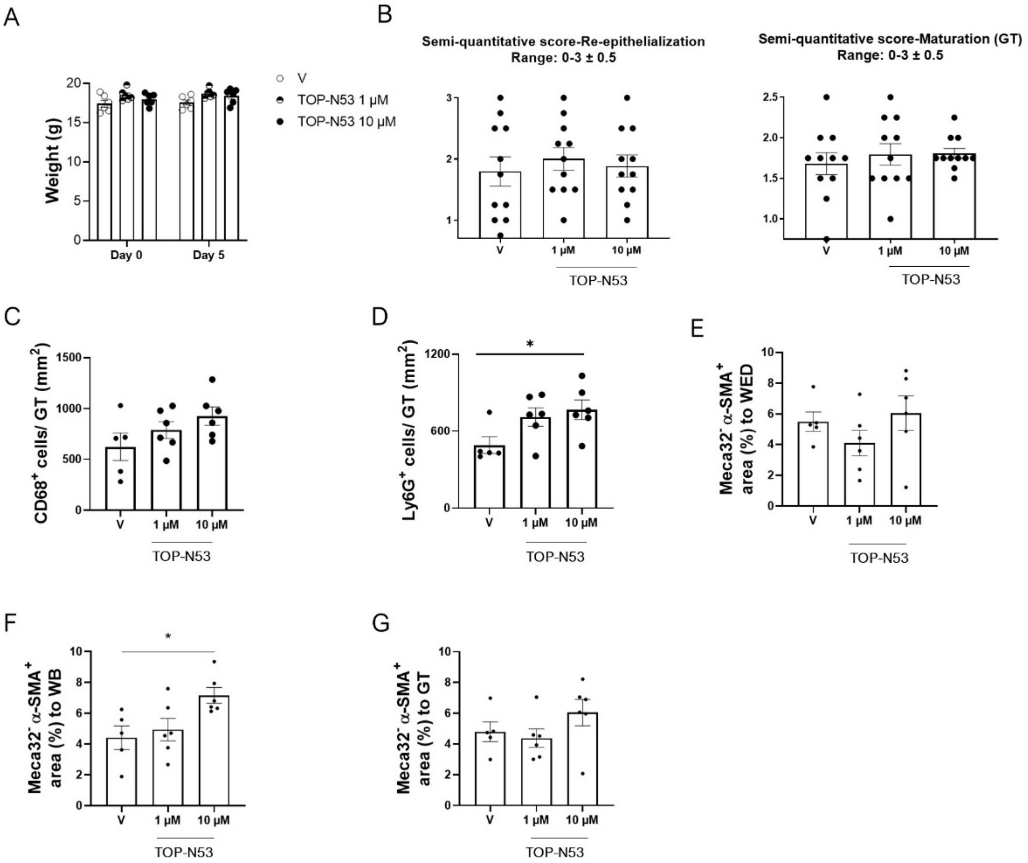

Figure S1. The effect of TOP-N53 liquid hydrogel formulation on wound healing in healthy C57BL/6J mice. **(A)** Body weight of healthy mice treated with vehicle (V) or TOP-N53 liquid hydrogel formulation at concentrations of 1 μM or 10 μM prior to (day 0) and at day 5 post-injury. N=6 per treatment group. **(B)** Semi-quantitative wound scoring for wound re-epithelialization (left) and maturation (right) performed by blinded investigators using sections from 5-day wounds treated with vehicle or TOP-N53 formulation at concentrations of 1 μM or 10 μM. N=6 mice, n=11 wounds. $CD68^+$ (C) or $Ly6G^+$ (D) cells per mm^2 granulation tissue area in wounds treated with liquid hydrogel vehicle or TOP-N53 formulation at concentrations of 1 μM or 10 μM. N=5-6 mice. Percentage of α-SMA^+/$Meca32^-$ granulation tissue area at the wound edge (WED) (E) or in the wound bed (WB) (F) or the entire granulation tissue (GT) (G) of wounds treated with vehicle or TOP-N53 liquid hydrogel formulation at concentration of 1 μM or 10 μM. N=5-6 per treatment group. All bars represent mean +/- SEM. *$P \leq 0.05$; ordinary one-way ANOVA.

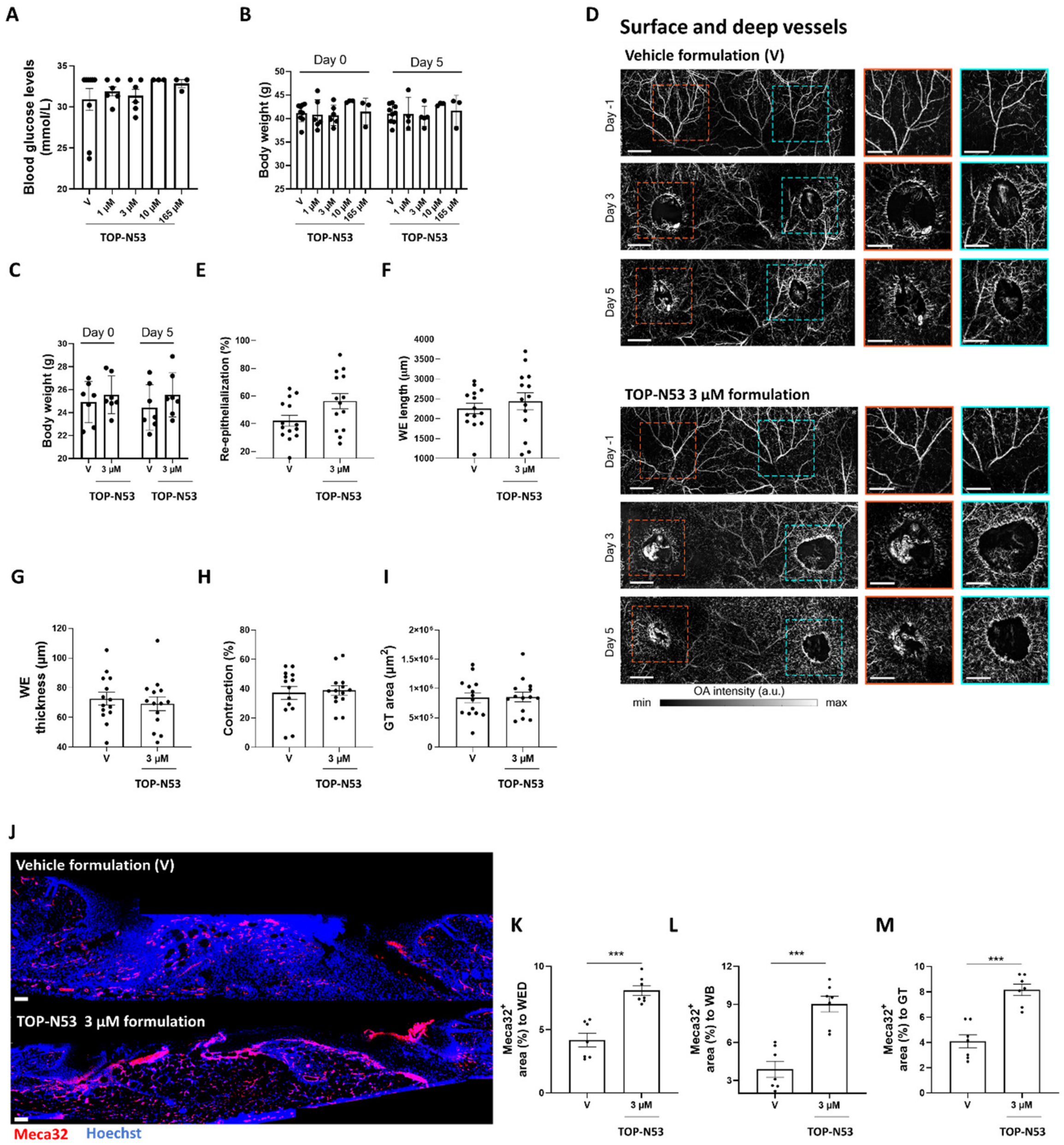

Figure S2. TOP-N53 liquid hydrogel formulation promotes wound vascularization. **(A)** Blood glucose levels and **(B)** body weight of diabetic mice treated with vehicle (V) or TOP-N53 liquid hydrogel formulation at concentrations of 1, 3, 10, or 165 µM prior to (day 0) and/or at day 5 post-injury. N=3-9 mice per treatment group. **(C)** Body weight of SKH-1 mice treated with vehicle or TOP-N53 liquid hydrogel formulation at a concentration of 3 µM prior to (day 0) and/or at day 5 post-injury. N=7 mice. **(D)** Representative longitudinal LSOM images acquired from non-wounded back skin (day -1) and wounds immediately after injury (day 0), or at 3- and 5-day post-injury of SKH-1 mice treated topically either with vehicle or TOP-N53 liquid hydrogel formulation at 3 µM and magnification of the data. Magnification bars: 2 mm (right panels) or 3 mm (left panel). The colormap represents optoacoustic signal intensity in arbitrary units (a.u.). **(E)** Percentage of wound re-epithelialization. N=7 mice, n=14 wounds. **(F)** Length and **(G)** average thickness (epidermis area/epidermis length) of the wound epidermis (WE). N=7 mice, n=14 wounds. **(H)** Percentage of wound contraction based on the initial wound length (5 mm). N=7 mice, n=14 wounds. **(I)** Area of granulation tissue (GT). N=7 mice, n=14 wounds. **(J)** Representative photomicrographs of wound sections stained for Meca-32 (red) and counterstained with Hoechst (blue). Magnification bars: 100 µm. Percentage of granulation tissue area at the wound edge area (WED) **(K)**, the wound bed area (WB), **(L)** or the total granulation tissue area **(M)** stained positive for Meca32. N=7 mice. All bars represent mean +/- SEM. ***P ≤ 0.001; Mann-Whitney U test (for comparison of vehicle formulation and TOP-N53 formulation-treated wounds); ordinary one-way ANOVA.
